# Management of Cryptococcosis: How Are We Doing?

**DOI:** 10.1371/journal.pmed.0040047

**Published:** 2007-02-06

**Authors:** John R Perfect

## Abstract

Perfect discusses the implications of a national prospective study (the "CryptoA/D" study) of the factors influencing clinical presentation and outcome of patients with cryptococcosis.

In a new research article published in *PLoS Medicine*, Francoise Dromer and colleagues [1] report on a French national prospective study (the “CryptoA/D” study) of the factors influencing clinical presentation and outcome of patients with cryptococcosis (an infection caused by the pathogenic fungus Cryptococcus neoformans). This is just the type of prospective study that is needed to help us understand how we are doing in the management of a life-threatening infection. Often our randomized, blinded, and controlled studies do not reflect actual outcomes in the general medical community, because study participants are biased by a series of entry criteria and other issues. Thus, prospective studies to collect real-time data during clinical management become important gauges in how successful we are and may help us define what we need to improve.

## The New Study

Dromer and colleagues present a multicenter surveillance of an entire country's experience of cryptococcosis management over a four-year period. Their study of 230 patients came to three major conclusions.

**Figure 1:**
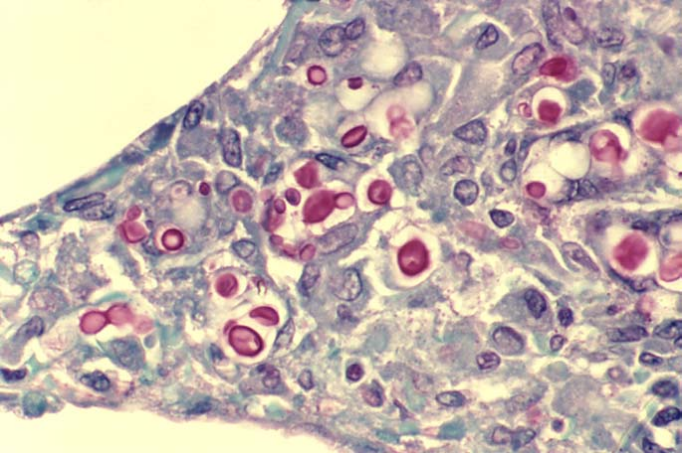
Cryptococcosis of the Lung in a Patient with AIDS Histopathology of lung shows widened alveolar septa containing a few inflammatory cells and numerous yeasts of the fungal pathogen C. neoformans. The inner layer of the yeast capsule stains red. Cryptococcosis is transmitted through inhalation of airborne yeast cells and/or biospores. At risk are the immunocompromised, especially those with HIV infection. Photo: CDC/Dr. Edwin P. Ewing, Jr.

First, from the initial assessment, higher severity of illness and poorer outcome occurred in: (1) men; (2) HIV-positive patients; and (3) patients infected with serotype A strains. These three factors capture three potential issues of: genetic susceptibility and/or hormonal influences, host immunity, and strain virulence or microbial fitness in cryptococcosis. Many years ago, the importance of male sex and outcome of cryptococcosis was observed in controlled animal experiments [2]. In cryptococcosis, host immunity influences the production of disease and disease outcome, and cell-mediated immunity is always the focal point in this infection. The host immune system, which can be altered by HIV infection or high-dose corticosteroids, controls the appearance and severity of cryptococcal infection (Figure 1). Finally, the particular invading cryptococcal strain may have some impact on disease. In animals cryptococcal strains vary in their ability to produce disease with the same inoculum, and in the Dromer et al. human study serotype A strains were more likely than serotype D strains to produce severe disease. Further validation of the importance of specific strains on disease is illustrated by the large outbreak of Cryptococcus gattii on Vancouver Island, Canada, in immunocompetent hosts with a probable recombinant hypervirulent strain [3].

The second finding of this study is the direct mycological impact of treatment. With the use of an endpoint at two weeks of mycological failure (defined as at least one cultured sample from the patient containing viable C. neoformans), the researchers found that failure was associated with initial dissemination of infection, high serum antigen titers, and the lack of flucytosine use during induction therapy. It is clear that patients with a high burden of organisms are at risk for failure, and initial cultures of several body sites and antigen titers can help identify these high-risk patients. This study also continues to validate the strategy of using the combination therapy of amphotericin B plus flucytosine [4]. The benefits of adding flucytosine are related to its faster “sterilization” of tissue and fluids [5], and the combination approach is associated with fewer relapses compared with monotherapy [6].

Third, this study identified the three factors that contributed to reduced three-month survival: (1) abnormal neurology, (2) abnormal brain imaging at baseline, and (3) underlying hematological malignancy. When there is more disease produced by infection, the outcome is worse and our vigilance in care must be even greater. On the other hand, for many of these invasive fungal infections the final arbiter of success will be the underlying disease and unfortunately, this issue may be difficult to control. For example, several decades ago Kaplan et al. noted that there was a poor prognosis for the combination of cryptococcosis and malignancy [7]; this fact has not changed.

## Implications of the Study

This study allows us to observe where we are in the management of cryptococcosis in the era of HAART (highly active antiretroviral therapy) and with access to current antifungal drugs and supportive care. Unfortunately, the failure rates remain substantial, with a mortality of about 12% at three months. Furthermore, there are undefined costs of care and morbidity from this infection. The question is: How do we do better?

## Next Steps

The answer is we must perform careful evidence-based studies to address a series of important clinical questions on patients at high risk for failure (see Box 1). We can then adjust our treatment guidelines to better fit the individual patient. This evidence-based directed research strategy will be good for the future of medicine but does not necessarily help the patient today. Therefore, the basic message of Dromer et al. rings as true as the message from the prognostic studies of cryptococcal meningitis with amphotericin B treatment in 1974 [8]. In the initial management of cryptococcosis, the assessment of the burden of yeasts in the host from its site of infection(s) to its quantity of yeasts determined through cultures, antigen loads, and radiographic appearances will give clinicians a prediction of the difficulties that they might face. Furthermore, the initial philosophy in the high-risk patient is to provide therapies that efficiently eliminate yeasts from the host. The sugar-coated killer whose sweetness sickens must be stripped away leaving only the underlying disease to deal with [9].

**Box 1. Questions Needing Precise Answers in the Management of Cryptococcosis**

1. What is the optimal management of increased intracranial pressure during meningitis?
2. How to specifically diagnose immune reconstitution syndrome (IRS) in cryptococcal meningitis and how to manage it?
3. During HIV infection and cryptococcal meningitis, when is it best to initiate HAART? For instance, is it different in resource-poor countries where azoles with less fungicidal activity have to be the inducing regimens for treatment?
4. Which is better to use: amphotericin B deoxycholate or lipid product of amphotericin B with flucytosine for induction therapy in cryptococcal meningitis?
5. Should we use the induction, clearance and suppression treatment strategy for all patients with cryptococcal meningitis and for how long should each stage be used? For instance, should patients with higher burden of yeasts have a longer treatment regimen at the induction and/or clearance stages?
6. When and how to use immune modulation during cryptococcal meningitis (gamma interferon for enhancement versus corticosteroids for depression of immunity in IRS)?
7. Do we need to serotype and perform in vitro susceptibility testing for each initial isolate?
8. When do we need to sample CSF when cryptococcus is isolated from an extraneural site?

In their study, Dromer and colleagues have provided us with a careful gauge of how we are doing with this life-threatening infection. In some respects, clinicians should be congratulated for their skills in caring for this deadly infection. On the other hand, there still remains a need for better strategies and treatments for cryptococcosis in all patients who suffer from the disease. Specific guidelines for management of cryptococcosis are helpful [10], and studies regarding risk factors provide insight, but until we have further evidence-based understanding, cryptococcosis treatment in some patients can have bedside nuances requiring judgments and adjustments one patient at a time.
